# Intravitreal Ranibizumab Versus Intravitreal Ranibizumab Combined with Posterior Subtenon Triamcinolone Acetonide in Diabetic Macular Edema

**DOI:** 10.14744/bej.2021.53315

**Published:** 2021-09-27

**Authors:** Gamze Karatas, Burak Erden, Akin Cakir, Selim Bolukbasi, Serkan Erdenoz, Bora Deniz Argon, Mustafa Nuri Elcioglu

**Affiliations:** 1. Department of Ophthalmology, Osmaniye State Hospital, Osmaniye, Turkey; 2. Department of Ophthalmology, University of Health Sciences, Okmeydani Training and Research Hospital, Istanbul, Turkey

**Keywords:** Diabetic macular edema, posterior subtenon triamcinolone acetonide, ranibizumab

## Abstract

**Objectives:**

This is a retrospective, comparative evaluation of the short-term efficacy and safety of intravitreal ranibizumab (IVR) and IVR combined with posterior subtenon triamcinolone acetonide (STA) in the treatment of diabetic macular oedema (DME).

**Methods:**

A total of 79 pseudophakic eyes of 57 patients with DME who underwent IVR injection treatment were examined retrospectively. All of the patients were treatment-naive. In the study group (STA+IVR), consisting of 30 eyes of 39 patients, the STA and IVR were administered in the first treatment session simultaneously, followed by 2 consecutive monthly IVR injections. In the control group (IVR only) comprised 40 eyes of 27 patients, 3 consecutive monthly IVR injections were administered. Patients with serous retinal detachment (SRD) according to optical coherence tomography images were identified in both groups for subgroup analyses. The primary outcome measures were changes in central macular thickness (CMT), best corrected visual acuity (BCVA), and the intraocular pressure (IOP) at 1, 2, and 3 months post-injection.

**Results:**

There was no statistically significant difference between the demographic characteristics of the patients’ baseline BCVA and CMT measurements (p>0.05). For the IVR group, the mean pre-treatment CMT and BCVA was 421.20±89.10 μm and 0.42±0.24 logMAR, respectively. After the third injection, the mean was 308.12±59.07 μm and 0.20±0.12 logMAR, respectively. The combined treatment group baseline measurements were 454.50±122.52 μm and 0.54±0.29 logMAR, respectively. After the third injection, the mean was 294.22±50.33 μm and 0.27±0.21 logMAR, respectively. The decrease was statistically significant for both groups (p=0.001). Comparison of the CMT within groups revealed a statistically significant difference in favor of the combined group after the second injection (p=0.017). There was no statistically significant difference in the BCVA gains between groups (p>0.05). Patients with SRD were evaluated as a subgroup, and at the first month, the mean gain in CMT was -71.63±57.98 μm in the control group and -123.61±93.46 μm in the study group (p=0.048). The required anti-glaucomatous treatment was statistically significant in the combined group (p=0.008).

**Conclusion:**

Both treatments provided improvement in BCVA and CMT and can be considered functional and anatomically effective treatment options for DME.

## Introduction

The most significant and common cause of visual impairment in patients with diabetic retinopathy (DR) is diabetic macular edema (DME) (1). In the past decades, special attention has been paid to the role of inflammatory mediators in the pathogenesis of DME. These mediators are known to be elevated secondary to retinal ischemia. Some inflammatory cytokines, such as interleukin-1 (IL-1) and IL-6, are thought to affect vascular permeability. The disintegrity in the tight junctions of the capillary endothelium secondary to the high levels of vascular endothelial growth factor (VEGF) may play a key role in DME pathogenesis (2). Hence, consecutive intravitreal anti-VEGF injections have been shown to improve visual acuity in several clinical trials (3, 4). In recent years, corticosteroid treatment – especially in the form of dexamethasone implant – has emerged again as an alternative treatment for DME with its anti-inflammatory, anti-VEGF, and antiproliferative effects (5, 6). Triamcinolone acetonide (TA), on the other hand, was a corticosteroid agent widely used for DME treatment in intravitreal or subtenon administrations before the introduction of anti-VEGF agents and dexamethasone implant. Both administration ways of TA, intravitreal TA (IVTA), and posterior subtenon TA (STA) were proven to be equally effective in the treatment of DME (7, 8).

A previous study reported that the efficacy of STA was similar compared to the same IVTA therapeutic dose of IVTA, and the reliability of STA was higher than IVTA (9). For this reason, we usually prefer STA in combination therapies at our retina department. In the current study, we analyzed our medical records retrospectively and searched for any additional beneficial effect of STA on the outcomes of IVR therapy in a comparative two-armed study design.

## Methods

### Ethics

This retrospective study has adhered to the tenets of the Declaration of Helsinki. The ethical approval of the study was obtained from the Institutional Ethical Board of the Okmeydani Research and Training Hospital (approval ID: 2017/753) in Istanbul, Turkey. Written informed consent had also been obtained from all the participants of the study.

### Patients

This study evaluated the medical records of 79 pseudophakic eyes of 57 treatment-naive patients who underwent IVR or IVR + STA combination treatment for DME at the Okmeydani Research and Training Hospital, Retina Department. All patients had type 2 diabetes mellitus. Thirty-nine eyes of 30 patients were included in the IVR alone (control) group and 40 eyes of 27 patients into the STA+IVR (study) group. DME was defined according to the central macular thickness (CMT) measurements higher than 260 μm. The patients were followed on a monthly basis and underwent detailed ophthalmologic examination including best-corrected visual acuity (BCVA) (Snellen), biomicroscopy, fundoscopy, intraocular pressure (IOP) measurement through Goldmann applanation, and spectral domain optic coherence tomography (SD-OCT) examination at each visit. In addition, all the patients underwent fluorescein angiography at their baseline visit. Forty-two eyes in the total study population had effective panretinal photocoagulation minimum 3 months previously to the study enrolment and no untreated peripheral ischemia. Twenty-two eyes had mild peripheral ischemia covering approximately <5 optic disc areas. In 15 eyes, no signs of retinal ischemia were detected on FA images. In the study (STA+IVR) group, the STA and IVR were administered in the first treatment session simultaneously followed by 2 consecutive monthly IVR injections. In the control (IVR only) group, 3 consecutive monthly IVR injections were administered.

Patients with a history of glaucoma, phakic eyes, any vitreomacular interface pathologies detected by SD-OCT, other vitreoretinal diseases and retinopathies than DR, severe peripheral ischemia covering greater than 5 optic disc areas or manifest foveal ischemia on FA, a history of vitrectomy, laser photocoagulation throughout the follow-up period, or anti-VEGF injections other than IVR were excluded from the study.

Patients with serous retinal detachment (SRD) from OCT images were identified in both groups to perform subgroup analyzes and determine whether responses to different treatment options were variable. The gains of IVR and IVR in combination with STA treatments on CMT and BCVA were evaluated in patients with SRD.

**Figure 1.**
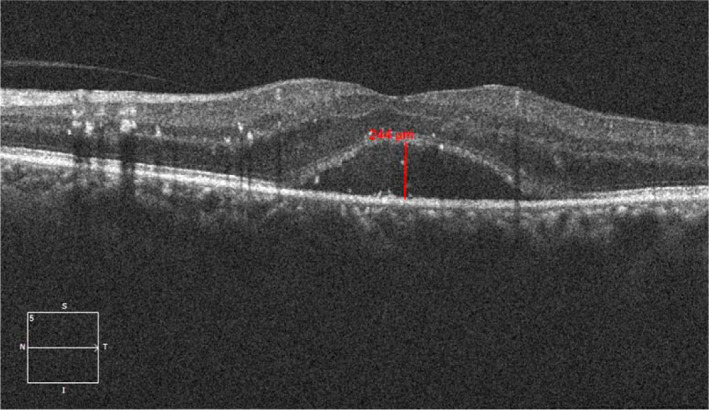
Measurement of SMD height with calipers in an OCT section.

SRD was defined as a shallow elevation of the retina and an optically clear space between the retina and retinal pigment epithelium. SRD elevation measurements were performed manually with the help of digital calipers from the retinal pigment epithelium to the ellipsoid zone through the OCT sections with the highest SRD. A measurement example is shown in Figure 1.

All the intravitreal injections were performed under topical anesthesia in the separate operating room. Informed consent was obtained from all patients before treatment. Under sterile conditions, a lid speculum was used and povidone-iodine was applied onto the ocular surface. In the control group, 0.5 mg ranibizumab (Lucentis, Genentech) was injected at 3.5 mm posterior to the corneoscleral limbus with a 30G needle. In the study group, a posterior subtenon TA injection was performed – at the first session – following the IVR injection. For this administration, conjunctiva and the Tenon capsule were incised in the inferonasal quadrant, approximately 6–8 mm posterior to the limbus using smooth microforceps and conjunctival scissors. Next, a 23-gauge curved blunt subtenon cannula was introduced through this peritomy, then, 40 mg/1 mL TA was injected into the posterior subtenon space. OCT (Cirrus SD-OCT Model 4000, Carl Zeiss Meditec, Dublin, California, USA) was used to determine the presence of SRD and the mean central value of CMT at each visit. Moreover, SRD elevation values were measured manually by two independent researchers, and the mean values were taken into the dataset.

All the patients underwent standard ophthalmic examinations at baseline and 1, 2, and 3 months postoperatively. The examinations included BCVA, slit-lamp biomicroscopy, Goldmann applanation tonometry, fundoscopy, and SD-OCT. The BCVA was measured with a Snellen chart, and the decimal visual acuity was converted to the logarithm of the minimal angle of resolution (LogMAR) units for statistical analyses. During follow-ups, anti-glaucomatous treatment was initiated in the patients with IOP higher than 21 mmHg or >5 mmHg than baseline and other complications were recorded.

### Statistical Analyses

The Statistical Package for the Social Sciences for Windows version 21 software was used to evaluate the data obtained in the study. In addition to the descriptive statistical methods, a paired t-test was used for intragroup comparisons of the parametric parameters and a Wilcoxon sign test was used if the parameters were non-parametric for intragroup comparisons. To compare the distributions of two or more variables related to each other, the Friedman test was used. The change in CMT and BCVA overtime was investigated using repeated measures of analysis of variance. Multivariate linear regression analysis was performed to determine the predictive factors for the improvement of CMT and BCVA. p<0.05 was considered statistically significant.

## Results

A total of 79 pseudophakic eyes of 57 patients were included in this study. When the demographic and clinical features of the patients were examined in all groups and subgroups, there was no statistically significant difference between the groups (p>0.05). Table 1 shows the demographic and clinical characteristics of the patients.

**Table 1.** Demographic and clinical characteristics of the study and control groups

	**Control Group**	**Study Group**	**p**
Age (years±SD)	62.56±8.71	62.87±8.72	0.764
Gender (female/male)	15 (50%)/15 (50%)	15 (55%)/12 (45%)	0.568
Side (right/left)	20/19	22/18	0.742
Duration of DM (years±SD)	14.6±5.27	12.9±5.13	0.200
Insulin usage, n (%)	18 (46)	17 (43)	0.582
Hypertension, n (%)	15 (39)	14 (35)	0.314
HBA1C Levels, (%)	7.9±1.7	8.1±1.7	0.911
Presence of SRD, n (%)	19 (48)	18 (45)	0.928
Baseline CMT (μm)	421.20±89.10 μm	454.50±122.52 μm	0.399
Baseline BCVA	0.42±0.24 logMAR	0.54±0.29 logMAR	0.075

DM: Diabetes mellitus; SRD: Serous retinal detachment; CMT: Central macular thickness; BCVA: Best-corrected visual acuity; SD: Standard deviation.

In the control group, the CMT significantly decreased from 421.20±89.10 μm at baseline to 358.71±75.78 μm, 332.20±68.19 μm, and 308.12±59.07 μm at the 1-, 2-, and 3-month visits, respectively. In the study group, the CMT significantly decreased from 454.50±122.52 μm at baseline to 334.60±73.88 μm, 301.92±51.95 μm, and 294.22±50.33 μm at the 1-, 2-, and 3-month follow-ups, respectively. The reduction in CMT for both groups was statistically significant (p<0.001).

The mean BCVA was 0.42±0.24 logMAR in the control group and 0.54±0.29 logMAR in the study group. It was measured as 0.27±0.15 logMAR, 0.24±0.13 logMAR, and 0.20±0.12 logMAR at the 1-, 2-, and 3-month visits in the control group and 0.35±0.24 logMAR, 0.31±0.22 logMAR, and 0.27±0.21 logMAR at the 1-, 2-, and 3-month visits in the study group. In both groups, the change in BCVA was statistically significant (p<0.001).

**Figure 2.**
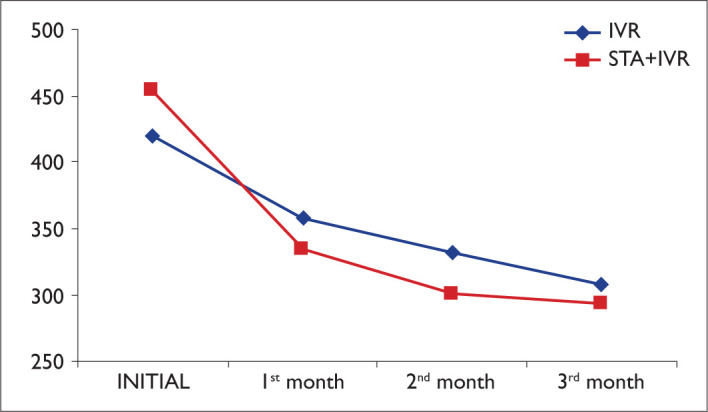
The distribution of CMTs over time among groups.

In intergroup comparison, the baseline CMT in the control group was 421.20±89.10 μm and 454.50±122.52 μm in the study group (p=0.399). In the 1^st^ month, the mean CMT was decreased to 358.71±75.78 μm in the control group and 334.60±73.88 μm in the study group with no significant intergroup difference (p=0.165). In the 2^nd^ month, the mean CMT was 332.20±68.19μm in the control group whereas it was 301.92±51.95 μm in the study group. The reduction in CMT at month 2 was found significantly higher in the study group compared with the control group (p=0.017). At the 3^rd^ month, the mean CMT values were 308.12±59.07 μm in the control group and 294.22±50.33 μm in the study group. These reductions were not statistically significant among the groups (p=0.303). Figure 2 shows the distribution of CMTs overtime according to groups.

Considering the changes in BCVA overtime among groups, no statistically significant difference was observed between the two groups. The changes in BCVA overtime are summarized in Table 2.

**Table 2.** The distribution of changes in BCVA among the groups

	**Group IVR**	**Combined Group**	**p**
Baseline BCVA	0,42±0.24 logMAR	0.54±0.29 logMAR	0.075
BCVA at the 1^st^ month	0.27±0.15 logMAR	0.35±0.24 logMAR	0.213
BCVA at the 2^nd^ month	0.24±0.13 logMAR	0.31±0.22 logMAR	0.135
BCVA at the 3^rd^ month	0.20±0.12 logMAR	0.27±0.21 logMAR	0.138

BCVA: Best-corrected visual acuity; IVR: Intravitreal ranibizumab.

**Figure 3.**
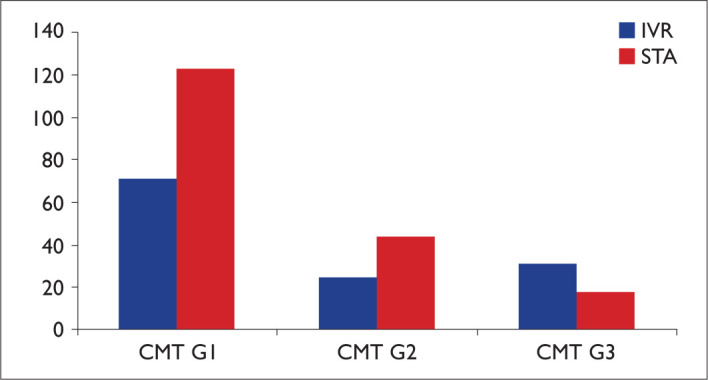
Mean CMT gain in SRD patients among groups (CMT G1: CMT gain at the 1^st^ month, CMT G2: CMT gain at the 2^nd^ month, CMT G3: CMT gain at the 3^rd^ month).

In a subgroup analysis, patients with SRD were evaluated as a subgroup; at the 1^st^ month, the mean gain in CMT was −71.63±57.98 μm in the control and −123.61±93.46 μm in the study group. The decrease of CMT values in the study group was significantly higher at the 1^st^ month visit (p=0.048). In the 2^nd^ month, the mean gain in CMT was −25.89±37.73 μm in the control group and −44.44±51.83 μm in the study group with no significant difference among the groups (p=0.218). At the 3^rd^ month, the mean gain in CMT was −31.10±33.20 μm in the control and −18.22±33.06 μm in the study group with no significant difference (p=0.143). The distribution of the mean CMT gain overtime among the groups is shown in Figure 3.

Considering the gains in BCVA in patients with SRD only, no statistically significant difference was found between the two groups. The gains in BCVA overtime are shown in Table 3.

**Table 3.** The distribution of visual gains in SRD patients of control and study groups

	**Control Group**	**Study Group**	**p**
BCVA gain at the 1^st^ month	0.20±0.16 logMAR	0.22±0.19 logMAR	0.760
BCVA gain at the 2^nd^ month	0.04±0.07 logMAR	0.06±0.11 logMAR	0.494
BCVA gain at the 3^rd^ month	0.04±0.05 logMAR	0.03±0.06 logMAR	0.442

SRD: Serous retinal detachment; BCVA: Best-corrected visual acuity.

In the total study population, the IOP elevation >5 mmHg than baseline or 21 mmHg was observed in only one patient in the control group but five patients in the study group. The IOP elevations were treated with topical anti-glaucomatous therapy in all cases and no surgical treatment was required. However, the incidence of IOP elevation was statistically significant in the study group compared to the control group (12% vs. 5%; p<0.008).

## Discussion

In the past decade, intravitreal anti-VEGF agents (aflibercept, bevacizumab, and ranibizumab) have begun to play a key role in the treatment of DME and they all provide positive advances in the prognosis of DME patients. However, combination therapies are reserved for poor or non-responder cases besides switching to a different anti-VEGF agent or available corticosteroids. The most logical way of combination therapies is to combine two different agents acting on two different pathways in the pathogenesis of DME. In the current study, we aimed to reveal our results of anti-VEGF and corticosteroid combination in an attempt to search for any additional gain among the DME patients.

Recently, Wang et al. reported their results of such a comparative study. They applied intravitreal bevacizumab (IVB) (1.25 mg/0.5 mL) to one group of DME patients, and in the other group, they combined IVB (1.25 mg/0.05 mL) with IVTA (2 mg/0.05 mL). They reported the short-term results (3 months) and the changes in CMT and BCVA compared to the baseline were statistically significant in both groups (p<0.001).

However, no significant difference was found between the two groups in terms of CMT and BCVA (10). Similarly, changes in CMT and BCVA were found to be statistically significant in both groups at the 3^rd^ month when compared to the baseline (p<0.001), and there was no statistically significant difference between the two groups in terms of BCVA and CMT improvement. Although the administration way of triamcinolone in our study is posterior subtenon rather than intravitreal, our short-term results are consistent with the results of this previous report.

Eris et al. (11) reported the comparative 6-month results of IVR therapy combined with STA versus IVR therapy alone in patients with resistant DME. They found the combination therapy statistically significant in terms of both BCVA and CMT changes, with the greatest difference recorded at the 1^st^ month visit of their 6 months follow-up. We also found the greatest difference in favor of our combination therapy group in means of anatomical results and it was observed at the 2^nd^ month visit, although there was no statistically significant difference between groups at our final 3^rd^ month visit. The primary difference between studies was that our study population consisted of treatment-naive individuals. Therefore, it is not surprising that the naive patients of our study population responded equally well to both treatment regimens. Another difference between Eris et al.’s and ours was the timing of the STA. In our study, STA injection was performed at the same session as the first IVR injection, while it was performed 10 days later in the Eris et al.’s study. We believe that performing the STA injection simultaneously with the IVR treatment is less time consuming and decreasing the number of visits. In another study, IVR was combined with an intravitreal dexamethasone implant for resistant DME cases. At the end of the 6-month follow-up, there was no statistically significant difference in terms of BCVA. However, there was a significant difference in favor of combination therapy in terms of CMT (12). These visual results in Maturi et al.’s report might be associated with the cataract progression in the phakic patients secondary to the intravitreal dexamethasone implants. Although we excluded the phakic eyes from our study population to eliminate a possible cataract progression on our visual results, there was no statistically significant difference in the improvement of BCVA between the two of our groups.

In a previous study, Kim et al. (13) searched for the aqueous concentrations of angiogenic and inflammatory cytokines in DME patients. In an attempt to associate the OCT findings and the aqueous cytokine levels, they divided the study population into three groups based on their OCT patterns: SRD, diffuse retinal thickening (DRT), and cystoid macular edema (CME). They found that inflammatory cytokines were found in a higher ratio in SRD patients than in DRT cases, but the ratio of angiogenic cytokines was comparable in both groups. In our study, we analyzed the SRD patients in a subgroup analysis, to find out if STA had any additional effect on CMT and BCVA in this subgroup. This subgroup analysis revealed an early – 1^st^ month – and significant anatomical gain in SRD patients under combination therapy. This finding bases possibly on the rapid effects of corticosteroids on inflammatory cytokines induced SRD. Although the decrease in SRD measurements continued in the 2^nd^ and 3^rd^ months, there was no significant difference between the two groups at the 3^rd^ month visit.

Ercalik et al. (14) reported in a DME population with SRD that they achieved a better improvement in CMT in the IVR + STA combination study group than their IVR only control group. To analyze this beneficial effect of STA, Yu et al. (15) compared the changes in the aqueous cytokine levels after IVB with those after combined IVB + STA injection in DME patients. They showed that monocyte chemotactic protein-1, platelet-derived growth factor-AA, IL-8, and VEGF levels decreased significantly in the IVB + STA group but only the VEGF level decreased in the IVB group (p=0.001) . These results are consistent with our findings. We believe that the rapid improvement of CMT after the first combined session was due to the anti-inflammatory effect of the triamcinolone in the combination. This rapid efficacy is also consistent with the duration of STA action and it is especially effective in patients with SRD and high inflammatory cytokines. Özdemir et al. (16) also reported that IVTA treatment is effective in patients with SRD and explained its efficacy with its anti-inflammatory effect.

The incidence IOP elevation in our study group was significantly higher compared to the control group (p<0.008). Ozdek et al. (9) investigated in a comparative study the therapeutic effects of IVTA and STA in DME patients, they reported that both administration ways were significantly and equally effective in the treatment of macular edema that they emphasized that STA therapy was more reliable than IVTA, especially in means of IOP changes. In our study, we found that the elevation of IOP was 12% in the STA combined study group and 2.5% in the IVR only control group. Compared to the previous studies, where IOP elevations or ocular hypertension after STA injection were reported between 30.7% and 16.2%, our results reflect a more moderate profile (17, 18). In our study, we chose particularly posterior subtenon administration of triamcinolone for its similar effectiveness, and it causes less frequently IOP elevations.

The most significant limitation of our study is the lack of randomization due to its retrospective nature. However, we deem this deficiency eliminated due to the age and gender distribution of the patients, comparable baseline CMT and BCVA values, and follow-ups and treatments were performed by the same physicians. We believe that there is a need for prospective randomized clinical trials with longer follow-up periods and a higher number of patients to search for the effectiveness of anti-VEGF and corticosteroid combination therapies.

## Conclusion

According to the results of our study, both treatments are effective on BCVA and CMT. Since there is no significant difference in the results of both treatment groups, we think that additional steroid administration is not required routinely. However, when we aim for rapid anatomical success in patients with SRD where inflammation is at the forefront (it may affect functional success in the long term), we find it useful in addition to IVR treatment, with a single session steroid application.

## Disclosures

### Ethics Committee Approval:

Okmeydani Training and Research Hospital Ethics Committee, protocol number: 48670771-514.10, Date: 07/11/2017.

### Peer-review:

Externally peer-reviewed.

### Conflict of Interest:

None declared.

### Authorship Contributions:

Involved in design and conduct of the study (GK, SB, AC, BE, SE); preparation and review of the study (GK ,BE, ME); data collection (GK, BE, SB, SE, BDA); and statistical analysis (AC).
